# Diagnostic concordance of dermatopathology and PCR in differentiating eczema from psoriasis

**DOI:** 10.1111/jdv.70388

**Published:** 2026-03-12

**Authors:** Andrea Schmitt, Susanne Proksch, Ludwig Gutzweiler, Sandra Roth, Marcella Engler, Cornelia S. L. Müller, Andreas Volz, Andreas W. Arnold, Monika Šedivcová, Adriana Bernklauova, Miroslav Dura, Denisa Kacerovska, Katja Technau‐Ihling, Christian Ihling, Christiane Rakozy, Wiebke Pruessmann, Thomas Leibing, Maria Isabel von Eichborn, Johannes Kern, Elisabeth Oms, Stefanie Eyerich, Kilian Eyerich, Helmut Laaff, Natalie Garzorz‐Stark, Kristin Technau‐Hafsi

**Affiliations:** ^1^ Department of Dermatology and Venereology, Faculty of Medicine University of Freiburg Freiburg Germany; ^2^ Dermagnostix GmbH Freiburg Germany; ^3^ Center for Histology, Cytology and Molecular Diagnostics Trier Germany; ^4^ Saarland University Homburg/Saar Germany; ^5^ Dermpath Basel GmbH Basel Switzerland; ^6^ Biopticka Laborator Pilsen Czech Republic; ^7^ Department of Pathology, Faculty of Medicine in Pilsen Charles University Pilsen Czech Republic; ^8^ Optipath Pathologie Frankfurt Germany; ^9^ Department of Dermatology, Allergology and Venereology University Medical Center Schleswig‐Holstein Luebeck Germany; ^10^ MVZ Dermatopathologie Prof. Kind Offenbach Germany; ^11^ Department of Dermatology University Hospital Regensburg Regensburg Germany; ^12^ Department of Dermatology Alfred Health and the School of Translational Medicine Monash University Melbourne Australia; ^13^ MVZ Laaff GmbH Freiburg Germany; ^14^ Institute for Immunodeficiency, Center for Chronic Immunodeficiency Medical Center‐University of Freiburg, Faculty of Medicine, University of Freiburg Freiburg Germany

**Keywords:** CCL27, dermatopathology, eczema, inter‐observer variability, molecular classifier, non‐communicable skin disease, NOS2, psoriasis, qPCR

## Abstract

**Background:**

Targeted treatments for non‐communicable chronic inflammatory skin diseases like eczema and psoriasis offer significant potential for effective therapy. However, therapeutic success requires an accurate diagnosis, which is challenging due to their overlapping clinical and histological features.

**Objectives:**

We aimed at assessing the diagnostic performance of both a manual (MC) and fully automated (PsorX‐LabDisk) quantitative real‐time polymerase chain reaction (RT‐qPCR) test based on the expression of *NOS2* and *CCL27* compared with conventional dermatopathological evaluation in differentiating psoriasis from eczema.

**Methods:**

Seventy‐three formalin‐fixed, paraffin‐embedded skin samples of psoriasis and eczema were randomly selected and evaluated histopathologically (H&E‐stained sections) by 14 dermatopathologists to assess inter‐observer variability, quantified using Cohen's and Fleiss' *κ*. To confirm that the observed variability was not cohort‐ or rater‐specific, a validation cohort (*n* = 72) from an independent institution was assessed by three dermatopathologists under identical conditions. For molecular analysis, both manual (MC) and automated *NOS2/CCL27*‐based RT‐qPCR (PsorX‐LabDisk) workflows were applied. Diagnostic performance (sensitivity, specificity, accuracy) of histopathological and molecular analyses was determined against reference diagnoses.

**Results:**

Dermatopathological evaluation demonstrated only fair agreement (Fleiss' *κ* = 0.31) in both study and validation cohort. The mean diagnostic accuracy of dermatopathology was 76.9%, with a sensitivity of 70% and specificity of 81.6%. In comparison, MC and the PsorX‐LabDisk achieved sensitivities of both 92.9%, specificities of 82.2% and 84.4% and accuracies of 87.7% and 86.3%, respectively. In diagnostically ambiguous cases, molecular testing maintained high accuracy (>86%), clearly outperforming dermatopathology, which showed near‐random agreement and low accuracy (61.7%).

**Conclusions:**

Both MC and PsorX‐LabDisk provide a reliable, examiner‐independent complement to dermatopathology for differentiating psoriasis and eczema. By reducing diagnostic ambiguity, it enhances clinical confidence and supports more precise and timely therapeutic decisions in inflammatory skin disease management.

## Plain Language Summary

**What is the disease?:**

Psoriasis and eczema are common long‐term inflammatory skin diseases that cause symptoms such as itching, redness and scaling and can strongly affect quality of life. Although they are different conditions, they often look similar on the skin and under the microscope, making diagnosis difficult. An accurate diagnosis is essential because modern targeted treatments work only for specific diseases.

**Location of the study:**

This study was conducted in Germany and the Czech Republic.

**Aim of the study:**

The aim of this study was to determine how reliably psoriasis and eczema can be distinguished using standard microscope‐based pathology and whether a molecular test could improve diagnostic accuracy, especially in unclear cases.

**Methodology:**

We analysed skin biopsy samples from 73 patients with psoriasis or eczema in Germany. Fourteen expert dermatopathologists independently evaluated the samples to assess agreement between specialists. To confirm the findings, a second group of 72 samples from the Czech Republic was evaluated by three dermatopathologists. All samples were also analysed using a molecular test that measures the presence of two genes (NOS2 and CCL27) using a manual method and a fully automated test (PsorX‐LabDisk).

**Key findings:**

Pathologists often disagreed when diagnosing psoriasis versus eczema, particularly in difficult cases. The molecular test was more consistent and accurate than standard pathology and performed especially well when the diagnosis was unclear.

**Conclusions and practical implications:**

Molecular testing can support doctors when traditional diagnosis is uncertain. It may help patients receive appropriate treatment sooner and avoid unnecessary or ineffective therapies.

## Graphical abstract

Distinguishing psoriasis from eczema is challenging due to overlapping clinical and histological features. This study shows substantial inter‐observer variability among dermatopathologists and demonstrates that a NOS2‐/CCL27‐based molecular test, including a fully automated platform, provides more accurate and reproducible diagnoses, particularly in diagnostically ambiguous cases.

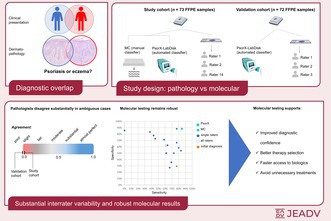

**Why was the study undertaken?**

- Psoriasis and eczema often show overlapping clinical and histopathological features, resulting in diagnostic uncertainty with important implications in the era of targeted therapies. This study was undertaken to assess how reproducible dermatopathological differentiation between these diseases is in routine practice and to determine whether molecular diagnostics can improve diagnostic accuracy, particularly in diagnostically ambiguous cases.

**What does this study add?**

- This study demonstrates substantial inter‐observer variability in dermatopathological diagnosis of psoriasis and eczema and shows that a molecular classifier based on NOS2/CCL27, including a fully automated qPCR platform, provides higher accuracy and reproducibility than conventional pathology, especially in ambiguous cases. It is the first study to benchmark both manual and automated molecular diagnostics directly against multi‐rater dermatopathological assessment.

**What are the implications of this study for disease understanding and/or clinical care?**

- The study highlights the limitations of morphology‐based diagnosis in inflammatory skin diseases and supports molecular diagnostics as an objective adjunct in challenging cases. Integrating molecular classifiers into routine practice may reduce misclassification, improve therapy selection and enable more precise use of targeted biologics, thereby enhancing patient outcomes and optimizing healthcare resource utilization.

## INTRODUCTION

Inflammatory skin diseases such as psoriasis and eczema are among the most prevalent chronic dermatologic conditions worldwide, with considerable implications for patient quality of life and healthcare resource utilization. Psoriasis affects 2%–3% of the global population, whereas atopic dermatitis (AD) is reported in up to 20% of children and 10% of adults, with rising prevalence in industrialized countries.^1^, ^2^, ^3^ While clinical presentation remains central to diagnosis, dermatopathological evaluation is frequently employed to confirm or differentiate these entities, particularly in atypical or treatment‐refractory cases.^4^, ^5^

Yet, even at the histomorphological level, psoriasis and eczema can share features such as epidermal hyperplasia, parakeratosis and lymphocytic infiltrates, complicating reliable distinction.^6^ As dermatopathology is qualitative and inherently subjective, inter‐observer variance is to be expected. Studies on inter‐observer agreement in chronic inflammatory skin diseases have largely addressed clinical severity scoring—such as PASI for psoriasis and EASI or SCORAD for AD.^7^, ^8^, ^9^ For these instrumentsintra‐ and interrater reliability ranges from fair to good. By contrast, comparable data for pathologist concordance in biopsy interpretation for these diseases are lacking. The absence of such data is notable given the central role of dermatopathology in the diagnostic process and thus its impact on therapeutic decision‐making. The growing repertoire of biologics targeting cytokines such as TNF, IL‐17, IL‐23 and IL‐4/13 has significantly improved outcomes in moderate‐to‐severe psoriasis and AD but also raised the stakes for accurate diagnostic classification.^6^, ^10^, ^11^, ^12^ Misclassification of a lesion—for instance, interpreting dermatitis as psoriasis or vice versa—may lead to inappropriate therapy and delayed access to effective biologics. It may also result in unnecessary exposure to systemic immunosuppressants, with implications for patient safety and public health costs.^13^, ^14^ Several strategies have been proposed to improve diagnostic consistency in inflammatory dermatoses. These include digital pathology platforms with AI‐assisted pattern recognition and the use of immunohistochemical adjuncts such as IL‐36γ to support or refine morphologic diagnoses.^15^, ^16^ Importantly, molecular diagnostics are increasingly recognized as a promising advancement in dermatopathology, particularly for differentiating between psoriasis and eczema in histologically ambiguous cases. Quaranta et al. showed that psoriasis and eczema exhibit distinct immune signatures, with psoriasis characterized by a Th17/IL‐17/IL‐36 profile and eczema by a Th2/IL‐4/IL‐13 signature with barrier dysfunction. Based on these findings, a molecular classifier using *NOS2* and *CCL27* gene expression was developed. This classifier accurately distinguished between psoriasis and eczema, including clinically ambiguous or initially misclassified cases.^17^, ^18^ Moreover, the assay proved effective in formalin‐fixed, paraffin‐embedded (FFPE) tissue, underscoring its suitability as a molecular tool for routine diagnostics.^14^ The present study addresses two key questions. First, how reproducible is dermatopathological differentiation of psoriasis and eczema among dermatopathologists? Second, can both a manual and a fully automated quantitative real‐time polymerase chain reaction (qPCR) assay, available as the PsorX‐LabDisk (Dermagnostix, Germany), enhance diagnostic accuracy compared with conventional pathology? By evaluating inter‐observer variability, we aimed to define the limitations of morphology‐based diagnosis. In parallel, we benchmarked molecular against dermatopathological performance to assess their added‐value in routine dermatopathology.

## MATERIALS AND METHODS

### Patient cohorts

This retrospective multi‐centre study included a study cohort from Germany and a validation cohort from the Czech Republic. The study cohort comprised 73 archived FFPE skin biopsy samples. These were randomly selected at the Department of Dermatology, Medical Center Freiburg from patients with diagnosed psoriasis or eczema (*n* = 28 psoriasis, *n* = 45 eczema) and selected based on the availability of sufficient clinical metadata. The study was designed as an exploratory comparative analysis focusing on inter‐observer variability and diagnostic concordance. The validation cohort included 72 archived FFPE skin samples collected at Biopticka Laborator (Pilsen, Czech Republic) from patients with clinically suspected psoriasis or eczema. Comprising a similar sample size as the study cohort, the validation cohort was used to confirm the reproducibility of interrater variability. The reference diagnosis of all patients in the study cohort was determined by three dermatologists based on all available metadata in a tertiary care university hospital. All available clinical metadata was considered, including initial clinical suspicion, clinical picture, family history, laboratory parameters, course of disease including therapeutic outcome and the dermatopathology report. In the validation cohort, a final reference diagnosis could not be established due to limited clinical information as expected for a referral laboratory. All patients gave written informed consent, and the study was approved by the local ethical committee of the University of Freiburg (project number 25‐1009‐S1) and the Faculty of Medicine in Pilsen (project number 122/25). Further patient characteristics are listed in Table 1.

**TABLE 1:** Patient characteristics of the study cohort and validation cohorts.

Characteristic	Study cohort total (*n* = 73)	Psoriasis (*n* = 28)	Eczema (*n* = 45)	Validation cohort (*n* = 72)
Age at biopsy (years)				
<35	10 (13.7%)	1 (3.6%)	9 (20%)	17 (23.6%)
35–54	13 (17.8%)	7 (25.0%)	6 (13.3%)	30 (41.7%)
55–74	41 (56.2%)	19 (67.9%)	22 (48.9%)	19 (26.3%)
>74	9 (12.3%)	1 (3.6%)	8 (17.8%)	6 (8.3%)
Sex				
Male	47 (64.4%)	15 (53.6%)	32 (71.1%)	39 (54.2%)
Female	26 (35.6%)	13 (46.4%)	13 (28.9%)	33 (54.8%)

*Note*: Age and sex distributions are shown for the study cohort and for the validation cohort. Percentages are calculated relative to the respective cohort sizes and are given in parentheses. The study cohort was further stratified according to the reference diagnosis (psoriasis and eczema), whereas for the validation cohort, psoriasis and eczema cases are presented as a combined group, as a final reference diagnosis could not be established due to limited clinical information.

### Dermatopathological assessments for analysis of inter‐observer reliability

In the study cohort, 73 samples were randomly selected, and their corresponding H&E‐stained slides were evaluated by 14 independent board‐certified dermatopathologists. The validation cohort included H&E‐stained slides of 72 skin samples, which were assessed by three independent board‐certified dermatopathologists. All evaluations were performed in a blinded manner, and the observers were unaware of each other's assessments to minimize bias. The original clinical suspicion was provided to all evaluators. Dermatopathological classification was performed using six categories: eczema, psoriasiform eczema, undetermined, eczematized psoriasis, psoriasis and diagnosis other than psoriasis or eczema. For subsequent analyses, these categories were consolidated into three groups: psoriasis, eczema and undetermined. Dermatopathological evaluation was performed independently of molecular testing results, and molecular analyses were conducted without access to dermatopathological classifications.

### Molecular assessment and comparison of molecular classifier result to reference diagnosis

Molecular testing was performed on FFPE skin biopsy specimens. Two workflows were applied: a manual qPCR approach and a fully automated polymerase chain reaction (PCR)‐based diagnostic platform (Dermagnostix LabDisk‐Analyer and PsorX‐LabDisk). Manual PCR was performed following the workflow, primers and probes previously described by Fischer et al.^14^ to ensure comparability with published protocols. Briefly, RT‐PCR assays for FFPE samples were carried out in 96‐well plates using a one‐step RT‐PCR master mix (Thermo Fisher Scientific, Waltham, MA, USA). Thermocycling and fluorescence detection were conducted on a qTower^3^ system (Analytik Jena, Germany), and amplification data were processed using qPCRsoft 4.1 (Analytik Jena). Expression levels of the target genes *NOS2* and *CCL27* were normalized to reference genes *TBP* and *SDHAF2* and analysed using the previously described algorithm.^14^ In parallel, the same FFPE samples were analysed using a fully automated, cartridge‐based PCR system (PsorX‐LabDisk, Dermagnostix, Germany). This system integrates RNA extraction, purification, reverse transcription, amplification and analysis into a single workflow. Results of PsorX‐LabDisk were available within approximately 2 h. Both the manual and automated PCR workflows generated an algorithm‐derived probability for each sample, classifying it as ‘psoriasis probable’ (probability > 55%), ‘undetermined’ (≥45% and ≤55%) or ‘psoriasis improbable’ (probability < 45%).

### Statistical analysis

All statistical analyses were performed using Microsoft Excel and Python 3 (Spyder 6 IDE), with the pandas, numpy and scikit‐learn. Inter‐observer agreement and diagnostic performance were assessed using Cohen's kappa (*κ*) for pairwise comparisons and Fleiss' kappa (*κ*) for multi‐rater agreement interpreted according to Landis and Koch. Samples were categorized as ‘ambiguous’ when diagnostic consensus of raters was low (<10/14 concordant raters in the study cohort or disagreement among ≥1/3 raters in the validation cohort). Diagnostic performance relative to the reference diagnosis was assessed by calculating sensitivity and specificity using a fourfold contingency table.

## RESULTS

### Dermatopathological assessment of psoriasis and eczema cases revealed substantial inter‐observer variability

**FIGURE 1:**
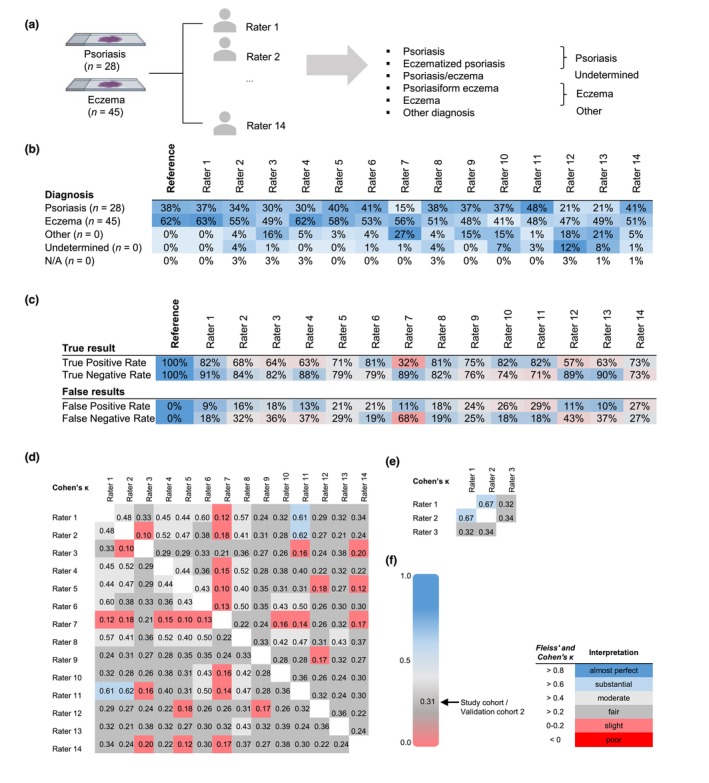
Interrater variability and diagnostic performance among dermatopathologists. (a) Study design: Skin biopsies from patients with psoriasis (*n* = 28) and eczema (*n* = 45) were independently evaluated by 14 dermatopathologists. Each rater assigned one of the following diagnoses: psoriasis, eczematized psoriasis, psoriasis/eczema, psoriasiform eczema, eczema or other diagnosis. For analysis, results were grouped into *psoriasis*, *eczema*, *undetermined* or *other*. (b) Distribution of diagnoses assigned by 14 dermatopathologists (Rater 1–14) compared to the reference diagnosis in the study cohort. (c) Diagnostic performance metrics for each rater. True positive rate (TPR) = proportion of psoriasis cases correctly identified as psoriasis; true negative rate (TNR) = proportion of eczema cases correctly identified as eczema; false positive rate (FPR) = proportion of eczema cases incorrectly diagnosed as psoriasis; false negative rate (FNR) = proportion of psoriasis cases incorrectly diagnosed as eczema. (d, e) Pairwise interrater agreement assessed using Cohen's *κ* for the study cohort (d) and the validation cohort (e). (f) Agreement as measured by Fleiss' *κ* and Cohen's *κ* with interpretation according to Landis & Koch.

Seventy‐three FFPE samples from psoriasis and eczema patients were evaluated by 14 independent dermatopathologists and assigned to psoriasis, eczema, undetermined or other diagnosis (Figure 1a). Thirty‐eight per cent of samples corresponded to the reference diagnosis of psoriasis (*n* = 28) and 62% to the reference diagnosis of eczema (*n* = 45). The overall distribution of diagnostic categories assigned by the individual raters roughly reflected the proportions of the reference diagnoses. This indicated no systematic bias towards either psoriasis or eczema in the study cohort (Figure 1b). However, when assessed at the level of individual samples, a considerable number of false‐positive and false‐negative classifications became evident (Figure 1c). Eczema was diagnosed correctly more frequently than psoriasis (82% ± 6.7% for eczema and 70% ± 13.7% for psoriasis). Psoriasis cases were more often underdiagnosed, with a mean false negative rate of 30% ± 13.7%, compared with a mean false positive rate of 18% ± 6.7% for eczema. True‐positive and false‐positive rates across raters varied substantially. Pairwise comparison of diagnostic performance independent of the reference diagnosis revealed Cohen's *κ* values ranging from only 0.12 to 0.62 (Figure 1d). To assess if low interrater consistency is a general phenomenon, we had three different pathologists evaluate a different cohort of *n* = 72 psoriasis and eczema samples in an identical experimental setting. Also in this cohort, Cohen's *κ* values ranged from 0.32 to 0.67 (Figure 1e). Overall inter‐observer agreement, as determined by Fleiss' *κ*, was fair to moderate (*κ* = 0.31 in both study and validation cohort), reflecting the intrinsic heterogeneity of dermatopathological interpretation in inflammatory skin diseases (Figure 1f).

### Both manual molecular classifier and automated PsorX‐LabDisk shows robust diagnostic performance

**FIGURE 2:**
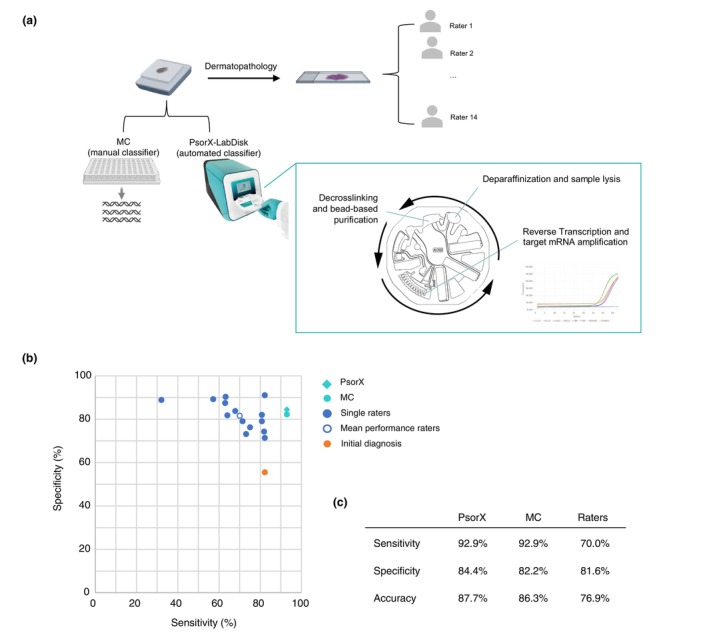
Diagnostic performance of the molecular classifier (MC), automated PsorX‐LabDisk system and dermatopathologists. (a) Performance Evaluation of Raters, Manual Classifier and PsorX‐LabDisk. PsorX‐LabDisk (in turquoise box) is a single‐use disposable microfluidic cartridge enabling fully automated processing of formalin‐fixed, paraffin‐embedded tissue samples. The integrated workflow, including automated deparaffinization and sample lysis, decrosslinking and bead‐based nucleic acid purification, reverse transcription and target mRNA amplification are schematically shown. The system performs all steps from sample input to quantitative real‐time polymerase chain reaction readout without manual intervention, enabling standardized, hands‐free molecular diagnostics. (b) Sensitivity and specificity of individual dermatopathologists (blue dots), mean performance of all dermatopathologists (white dot outlined in blue), initial clinical diagnosis (orange) and molecular assays (MC, turquoise; PsorX, turquoise square) in the study cohort. (c) Summary of diagnostic performance metrics showing sensitivity, specificity and overall accuracy for the manual molecular classifier (MC), fully automated PsorX‐LabDisk system and dermatopathological raters in the study cohort.

We next aimed to evaluate the diagnostic accuracy of each dermatopathologist by calculating sensitivity for the correct identification of psoriasis. Specificity was defined as the correct exclusion of psoriasis corresponding to a correct diagnosis of eczema and was assessed relative to the reference diagnosis in the study cohort (Figure 2a). Sensitivity values varied substantially among dermatopathologists, ranging from 32% to 82.1%, with an overall sensitivity of 70%. Specificity values were more consistent, ranging from 71.4% to 91.1% (mean specificity = 81.6%), corresponding to an overall diagnostic accuracy of 76.9% (Figure 2b,c). Given the high inter‐observer variability, we evaluated the performance of the molecular assay designed to distinguish psoriasis from eczema. The assay was performed in the study cohort using a commercially available fully automated system (PsorX‐LabDisk) and a manual qPCR workflow as the reference method. PsorX‐LabDisk is a cartridge‐based molecular diagnostic assay built on a centrifugal microfluidics (lab‐on‐a‐disk) platform, enabling fully automated sample processing from input to readout. The system integrates modules for sample preparation, nucleic acid purification, amplification (RT‐PCR) and detection, using a mix of lyophilized reagents and liquid reagents housed in stick‐packs allowing hands‐off operation (Figure 2a). Sensitivity of the conventional manual PCR workflow was 92.9%, specificity was 82.2%, and accuracy was 86.3%. Using the conventional manual PCR workflow as benchmark, the positive percent agreement sensitivity of PsorX‐LabDisk was 94.1%, negative percent agreement was 97.4%, and overall percent agreement was 95.9% compared to the manual PCR. These results show that the fully automated mode of operation sustains the high precision of molecular testing. More importantly, PosrX‐LabDisk demonstrated high and consistent diagnostic performance compared to the reference diagnoses, with a sensitivity of 92.9%, specificity of 84.4% and overall accuracy of 87.7% (Figure 2b,c).

### Molecular classification clearly outperforms dermatopathologists in ambiguous cases

**FIGURE 3:**
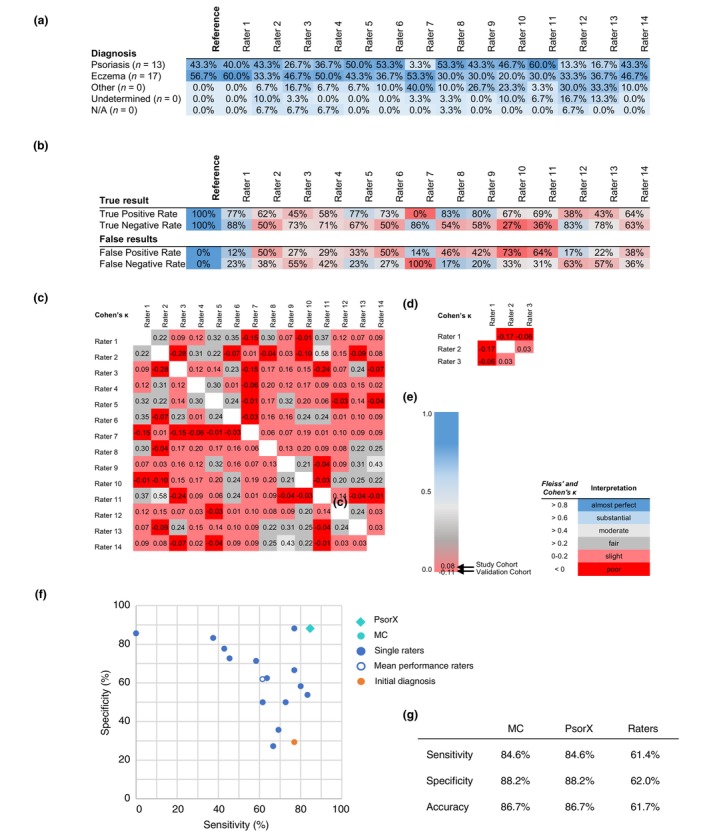
Interrater variability and diagnostic performance among dermatopathologists in ambiguous cases. (a) Distribution of diagnoses assigned by 14 dermatopathologists (Rater 1–14) compared to the reference diagnosis in the study cohort. (b) Diagnostic performance metrics for each rater. True positive rate (TPR) = proportion of psoriasis cases correctly identified as psoriasis; true negative rate (TNR) = proportion of eczema cases correctly identified as eczema; false positive rate (FPR) = proportion of eczema cases incorrectly diagnosed as psoriasis; false negative rate (FNR) = proportion of psoriasis cases incorrectly diagnosed as eczema. (c, d) Pairwise interrater agreement assessed using Cohen's *κ* for the study cohort (c) and the validation cohort (d). (e) Agreement as measured by Fleiss' *κ* and Cohen's *κ* with interpretation according to Landis & Koch. (f) Sensitivity and specificity of individual dermatopathologists (blue dots), mean performance of all dermatopathologists (white dot outlined in blue), initial clinical diagnosis (orange) and molecular assays (MC, turquoise; PsorX, turquoise square) in the study cohort. (g) Summary of diagnostic performance metrics showing sensitivity, specificity and overall accuracy for the manual molecular classifier (MC), fully automated PsorX‐LabDisk system and dermatopathology raters in the study cohort.

The reference diagnosis matched the initial clinical suspicion in only 48 of 73 cases (65.7%), indicating a cohort enriched for diagnostically challenging presentations and justifying biopsy. Given the high inter‐observer variability, we analysed a subgroup of ‘ambiguous cases’, defined by low rater concordance with PsorX. Thirty of 73 samples (43.3% psoriasis, 56.7% eczema) met this criterion (Figure 3a), In these cases, observer agreement was poor, with near‐equal rates of correct and incorrect diagnoses (FPR = 37% ± 18.5%, FNR = 40% ± 22.3%, Cohen's *κ* in study and validation cohort: −0.28 to 0.37 and −0.17 to 0.03, Fleiss' *κ* = 0.08 and –‐0.11 in study and validation cohort, Figure 3b–e). For dermatopathology, sensitivity values varied between 0% and 83.3%, with an overall sensitivity of 61.4%. Specificity values were more consistent, ranging from 27.3% to 88.2% (mean specificity = 62%), corresponding to an overall diagnostic accuracy of 61.7% (Figure 3f). In contrast, manual PCR and PsorX‐LabDisk maintained high diagnostic performance even in these challenging cases, with identical parameters for sensitivity of 84.6%, specificity of 88.2% and overall accuracy of 86.7% (Figure 3g).

### Representative cases illustrate the diagnostic value of the molecular classifier in unclear cases

**FIGURE 4:**
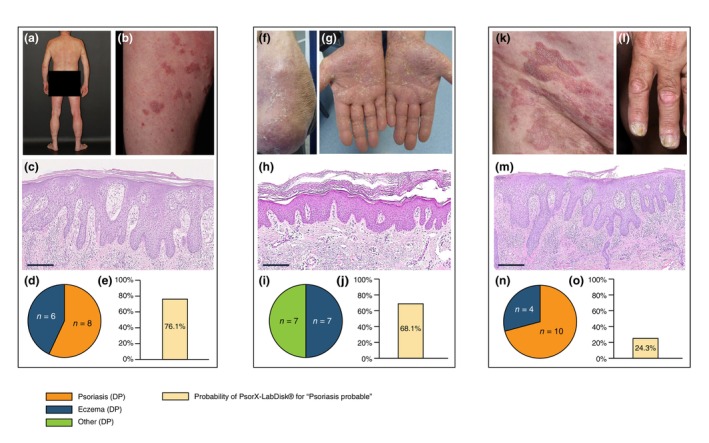
Representative cases illustrating diagnostic ambiguity between psoriasis and eczema and corresponding PsorX‐LabDisk results. (a–e) Patient 1 presented with nummular psoriasiform plaques clinically suggestive of both psoriasis vulgaris and nummular eczema (a, b) while histopathology showed psoriasiform hyperplasia (c). Eight of 14 dermatopathologists diagnosed psoriasis, whereas six favoured eczema (d). PsorX‐LabDisk indicated a 76.1% probability for psoriasis (e), consistent with therapeutic response to TNF inhibition. (f–j) Patient 2 presented with eczematous lesions at the elbow (f) and palmar pustular lesions (g) with rather eczematous features in histology (h). Diagnoses were evenly split between eczema and a diagnosis other than psoriasis or eczema in a biopsy from the elbow (i). PsorX‐LabDisk indicated a 68.1% probability for psoriasis (j), in line with clinical improvement under TNF‐ and IL‐17‐targeted therapy. (k–o) Patient 3 presented with psoriasiform plaques (k) and nail dystrophy (l) with dermatopathology showing psoriasiform changes (m). Ten of 14 dermatopathologists diagnosed psoriasis (n), whereas PsorX‐LabDisk yielded a 24.3% probability for psoriasis (o). The patient did not respond to various psoriasis therapies and was later diagnosed with mycosis fungoides. Scale bars = 200 μm. DP, dermatopathologists.

To further illustrate the potential of the molecular classifier to support diagnostic decision‐making, three representative patient examples highlight the clinical and histological overlap underlying diagnostic ambiguity between psoriasis and eczema. Patient 1 was a 63‐year‐old male who showed nummular psoriasiform plaques resembling both psoriasis vulgaris and nummular eczema. 6/14 dermatopathologists classified the lesion as eczema, whereas 8/14 voted for psoriasis. PsorX‐LabDisk showed a clear probability for psoriasis (76.1%) and accordingly, the patient responded to TNF‐inhibitor therapy (Figure 4a–c). Another 73‐year‐old male patient was initially suspected of pustular palmoplantar psoriasis or superinfected dyshidrotic hand eczema. Here, dermatopathological assessments were evenly split between eczema and a completely other diagnosis. In line with the patient's therapy response to TNF‐ and IL17 inhibition, PsorX‐LabDisk delivered a probability of 68.1% for the diagnosis of psoriasis (Figure 4d–f). A third patient, a 71‐year‐old male, showing psoriasiform skin lesions alongside psoriatic nail dystrophy, was predominantly interpreted as psoriasis (10/14 dermatopathologists). Interestingly, PsorX‐LabDisk showed a probability of only 24.3% for psoriasis. In fact, the patient did not respond to TNF or IL17 inhibition and had in the meantime died of mycosis fungoides (Figure 4g–i). Although PsorX‐LabDisk was developed only to differentiate between eczema and psoriasis, discrepancies between the PsorX‐LabDisk result and the clinical and dermatopathological examination may occur. In this case, discordant results may indicate a need to re‐examine the diagnosis.

## DISCUSSION

The clinical and dermatopathological features of psoriasis and eczema frequently overlap, leading to considerable rates of misclassification and diagnostic delay. Previous studies report that up to 15% of psoriasis cases are initially diagnosed as eczema, with misclassification rates reaching 50% in palmoplantar disease.^19^, ^20^ Diagnostic delays of several years are common in primary care, and misdiagnosis is more frequent in patients with skin of color.^21^, ^22^

Consistent with these findings, the initially suspected diagnosis in our study differed from the final reference diagnosis in 34.3% of cases. Given this clinical ambiguity, dermatopathological examination is commonly used to support diagnosis. However, our results demonstrate only fair to moderate inter‐observer agreement among dermatopathologists across two independent cohorts. Agreement was particularly low in diagnostically ambiguous cases underscoring the limitations of morphology‐based evaluation when inflammatory patterns overlap or remain subtle. This aligns with prior studies in other diagnostic fields, including melanocytic tumours, where almost 50% interrater variability persists even among experts.^23^ Thus, dermatopathology, while often regarded as the diagnostic gold standard, is itself subject to considerable variability. Nevertheless, several limitations of this study should be acknowledged. First, the reference diagnosis does not constitute an absolute gold standard but is based on retrospective expert clinical judgement without a fully standardized or blinded protocol. Although all available clinical information including treatment response and histopathology was considered, a potential bias may be introduced. As such, misclassification cannot be fully excluded, and all diagnostic performance metrics must be interpreted relative to this reference framework. Second, in our study only cases in which dermatologists considered a biopsy clinically necessary were included. This results in a study population enriched for diagnostically ambiguous presentations and limits generalizability to straightforward cases of psoriasis or eczema, in which inter‐observer agreement among dermatopathologists might be higher in more typical or unequivocal lesions. However, our cohort reflects real‐world scenarios in which diagnostic uncertainty is highest and adjunctive diagnostic tools are most needed. Third, the validation cohort included only three dermatopathologists, limiting direct quantitative comparison, but was intended to confirm the presence of inter‐observer variability.

Several molecular approaches have been proposed to complement traditional dermatopathological diagnosis and provide more objective discrimination between psoriasis and eczema. IL‐36γ immunohistochemistry and molecular profiling from tape strips have shown diagnostic potential.^24^, ^25^, ^26^ The most established tool for discriminating psoriasis from eczema on a molecular level has been introduced with a minimal gene‐expression classifier based on *NOS2* and *CCL27*. It was first introduced in 2014, and since then has been validated across multiple cohorts^17^, ^18^, ^27^, ^28^, ^29^, ^30^, ^31^, ^32^ and recognized in reviews and guideline summaries as a leading classifier in this space.^33^, ^34^, ^35^ In this study, both the manual classifier and its fully automated implementation (PsorX‐LabDisk, CE‐IVD) demonstrated higher accuracy and markedly greater reproducibility than dermatopathology, particularly in ambiguous cases. Although certain individual observers reached similar values in selected parameters, the molecular assay yielded markedly higher reproducibility and consistent classification across both cohorts. Notably, in the subcohort of diagnostically ambiguous cases, both the manual *NOS2/CCL27*‐based PCR classifier and the PsorX‐LabDisk assay maintained high diagnostic accuracy, demonstrating robust performance even under challenging conditions. Although the PsorX assay may theoretically yield ‘undetermined’ results near decision thresholds, no such cases occurred in this study. In general, undetermined results indicate biological overlap and should be interpreted as inconclusive, serving as a safeguard against overclassification and prompting further clinicopathological correlation. Over the past two decades, highly specific biologics have revolutionized the treatment of psoriasis and eczema.^12^ However, their pathway‐targeted mechanisms limit efficacy to defined patient subsets, emphasizing the need for precise diagnosis and therapy matching. In our study, the *NOS2/CCL27* classifier accurately reflected treatment response in patients with clinically and histologically ambiguous disease presentations. This suggests that in some instances, the molecular classifier may reveal its diagnostic accuracy only in retrospect when the true disease becomes evident. As psoriasis and eczema represent only a fraction of the inflammatory disease spectrum, future classifiers must extend to broader differential diagnoses. Recent studies have demonstrated the value of molecular profiling in inflammatory dermatoses. Seremet et al. identified immune gene expression modules that refine diagnosis and enable pathway‐based patient stratification, while Beaulieu et al. showed the diagnostic utility of a 15‐gene panel in inflammatory skin diseases.^36^, ^37^ Future studies directly comparing molecular approaches, as well as evaluating cost, workflow feasibility and turnaround time, are needed. These aspects were beyond the scope of the present study, which focused on diagnostic performance and reproducibility, and should be addressed in future prospective investigations. By reducing diagnostic ambiguity, molecular tools may nonetheless enhance clinical confidence, enable more precise, fast diagnostics and consequently reduce expensive trial‐and‐error therapy selection procedures.

## AUTHOR CONTRIBUTIONS

Andrea Schmitt: data collection, writing; Susanne Proksch: data collection and analysis, original draft preparation; Ludwig Gutzweiler: data analysis, review and editing; Sandra Roth: data analysis; Marcella Engler: data collection and data analysis; Cornelia S. L. Müller: data collection, review and editing; Andreas Volz: data collection, review and editing; Andreas W. Arnold: data collection, review and editing; Monika Šedivcová: data collection, review and editing; Adriana Bernklauova: data collection, review and editing; Miroslav Dura: data collection, review and editing; Denisa Kacerovska: data collection, review and editing; Katja Technau‐Ihling: data collection, review and editing; Christian Ihling: data collection, review and editing; Christiane Rakozy: data collection, review and editing; Wiebke Pruessmann: data collection, review and editing; Thomas Leibing: data collection, review and editing; Maria Isabel von Eichborn: data collection, review and editing; Johannes Kern: data collection, review and editing; Elisabeth Oms: data collection, review and editing; Stefanie Eyerich: data collection, review and editing; Kilian Eyerich: review and editing; Helmut Laaff: data collection, review and editing; Natalie Garzorz‐Stark: supervision, conceptualization, data analysis, review and editing; Kristin Technau‐Hafsi: supervision, conceptualization, data collection, review and editing.

## FUNDING INFORMATION

The study was funded by Dermagnostix GmbH.

## CONFLICT OF INTEREST STATEMENT

NG‐S, SE and KE are founders and shareholders of Dermagnostix GmbH and Dermagnostix R&D GmbH. NG‐S, SP, LG, SR and ME are employees of Dermagnostix GmbH. HL is a shareholder of Dermagnostix GmbH. All other authors AS, CSLM, AV, AWA, MS, AB, MD, DK, KT‐I, CI, CR, WP, TL, MIE, JK, EO and KT‐H declare no conflicts of interest related to this study. In this study, clinical and dermatopathological assessments were performed in a blinded manner and independently of molecular testing. Data analysis and interpretation were performed collaboratively by academic and industry‐affiliated authors.

## ETHICAL APPROVAL

The study was approved by the local ethical committee of the University of Freiburg (project number 25‐1009‐S1) and the Faculty of Medicine in Pilsen (project number 122/25).

## ETHICS STATEMENT

The patients in this manuscript have given written informed consent to the publication of their case details.

## Data Availability

The data that support the findings of this study are available from the corresponding author upon reasonable request.
